# Generating and evaluating a propensity model using textual features from electronic medical records

**DOI:** 10.1371/journal.pone.0212999

**Published:** 2019-03-04

**Authors:** Zubair Afzal, Gwen M. C. Masclee, Miriam C. J. M. Sturkenboom, Jan A. Kors, Martijn J. Schuemie

**Affiliations:** 1 Department of Medical Informatics, Erasmus University Medical Center, CA Rotterdam, Netherlands; 2 Janssen Research and Development LLC, Titusville, NJ, United States of America; University of Oxford, UNITED KINGDOM

## Abstract

**Background:**

Propensity score (PS) methods are commonly used to control for confounding in comparative effectiveness studies. Electronic health records (EHRs) contain much unstructured data that could be used as proxies for potential confounding factors. The goal of this study was to assess whether the unstructured information can also be used to construct PS models that would allow to properly deal with confounding. We used an example of coxibs (Cox-2 inhibitors) vs. traditional NSAIDs and the risk of upper gastro-intestinal bleeding as example, since this association is often confounded due to channeling of coxibs to patients at higher risk of upper gastro-intestinal bleeding.

**Methods:**

In a cohort study of new users of nonsteroidal anti-inflammatory drugs (NSAIDs) from the Dutch Integrated Primary Care Information (IPCI) database, we identified all patients who experienced an upper gastrointestinal bleeding (UGIB). We used a large-scale regularized regression to fit two PS models using all structured and unstructured information in the EHR. We calculated hazard ratios (HRs) to estimate the risk of UGIB among selective cyclo-oxygenase-2 (COX-2) inhibitor users compared to nonselective NSAID (nsNSAID) users.

**Results:**

The crude hazard ratio of UGIB for COX-2 inhibitors compared to nsNSAIDs was 0.50 (95% confidence interval 0.18–1.36). Matching only on age resulted in an HR of 0.36 (0.11–1.16), and of 0.35 (0.11–1.11) when further adjusted for sex. Matching on PS only, the first model yielded an HR of 0.42 (0.13–1.38), which reduced to 0.35 (0.96–1.25) when adjusted for age and sex. The second model resulted in an HR of 0.42 (0.13–1.39), which dropped to 0.31 (0.09–1.08) after adjustment for age and sex.

**Conclusions:**

PS models can be created using unstructured information in EHRs. An incremental benefit was observed by matching on PS over traditional matching and adjustment for covariates.

## Introduction

Electronic health records (EHRs) are primarily used for routine medical care, but secondary use of EHR data for observational research is becoming increasingly popular especially in studying of drug effects postmarketing [1]. In this era data is used to generate information on drug safety and effectiveness in a cost-efficient way and by exploiting actual care patterns, which differ largely from experimental settings [2–5]. In an experimental setting such as in randomized clinical trials, the choice for a treatment is randomized, which would take care of potential confounding by indication [6]. In actual care the treatment decision is usually influenced by measurable patient characteristics such as medical history, concomitant drug intake but also by personal prescriber preferences, which cannot be measured easily. This phenomenon of preferential prescribing is also known as channeling and may lead to confounding by indication [7,8]. A well-known example of channeling is the preference of doctors to prescribe selective cyclo-oxygenase-2 inhibitors (COX-2 inhibitors) over nonselective (ns) non-steroidal anti-inflammatory drugs (NSAIDs) to patients at risk of developing upper gastrointestinal bleeding (UGIB) [9,10], as the COX-2 inhibitors were developed on purpose to mitigate the GI effects of NSAIDs. Although clinical trials showed that COX-2 inhibitors are ‘safer’ than nsNSAIDs in relation to UGIB [11], observational studies showed no large differences between the rate of UGIB between COX-2 inhibitor and nsNSAIDs, possibly due to residual confounding by indications arising from channeling [12]. In order to obtain unbiased estimates in observational studies this confounding must be dealt with adequately. However, it is challenging to capture all relevant channeling factors in the EHR databases because information is not primarily recorded for research purposes. Moreover, relevant information may also be recorded in EHRs in an unstructured way [13,14].

Attempts to construct methods that deal with confounding have resulted in the propensity score method, the propensity score is an estimated conditional probability of receiving one particular treatment over another given a set of measured covariates [15], it can be regarded as a comprehensive way to look at channeling. Propensity score methods can be used to control for the unbalance between the treatment groups in order to estimate the comparative effectiveness of treatments [15]. Four different methods of using the propensity to reduce confounding have been described [16]: 1) matching on propensity score; 2) stratification on the propensity score; 3) inverse probability of treatment weighting using the propensity score; 4) and covariate adjustment using the propensity score. Typically, all variables related to either the outcome and/or exposure, are included in the propensity score model [17,18], sometimes these variables are not the exact confounding factors but proxies thereof [19]. Yet, identifying appropriate proxies in large EHRs is challenging. Schneeweiss *et al*. [20] proposed a high-dimensional propensity score (hd-PS) algorithm to empirically identify a large number of relevant covariates, with high prevalence, to control for confounding. In a case study on coxibs and NSAIDs using claims data in the USA, application of the hd-PS algorithm to control for confounding was found to produce an effect estimate for the risk of upper GI complications between coxibs vs. NSAIDs that was comparable to the one found in randomized trials [21]. The hd-PS model is constructed by using many covariates of which some could serve as proxies for unobserved factors that otherwise may not be considered. Typically, only structured information such as diagnostic or procedure codes that is available in the claims databases, are included in the model. Rassen et al. [22] evaluated whether adding two-word phrases, present in patients’ unstructured free-text data, to the propensity score model could improve validity of pharmacoepidemiology studies. Adjusting for two-word phrases resulted in an improvement in confounding adjustment. Electronic health records comprise much unstructured data and we propose that this information could also be used as proxies for potential confounding factors.

The aim of this study was therefore to assess whether unstructured text in EHRs can be used to construct a propensity score model that would allow to properly deal with confounding. We assessed the performance of propensity score models in addressing confounding by indication using as an example the association between selective COX-2 inhibitors and nonselective NSAIDs in relation to upper gastrointestinal bleeding.

## Methods

### Data source

We used data from the Dutch Integrated Primary Care Information database (IPCI) [23], a population-based general practice EHR database. This database contains prospectively collected routine care data representing real-life practice. In the Netherlands, all citizens are registered with a general practitioner (GP), who acts as a gatekeeper to secondary and tertiary medical care. IPCI contains information on more than 1.8 million patients from 340 GP practices. For each individual person, all relevant medical information from primary and secondary care is documented in the medical record. Apart from patient demographics, the recorded information in the EHRs contain medical notes (including symptoms, physical examination, assessments and diagnoses), drug prescriptions, laboratory results, referrals for hospitalization or specialist care, and hospital discharge summaries. In the IPCI database, drug prescriptions are recoded according to the Anatomical Therapeutical Chemical (ATC) classification for research purposes [24]. Diagnoses are coded according to the International Classification for Primary Care (ICPC) [25]. Almost 60% of the medical record are clinical narratives, which do not contain coded information, but contain important information such as patient-reported symptoms and notes from the GP.

### Selection of NSAID cohort

**Fig 1:**
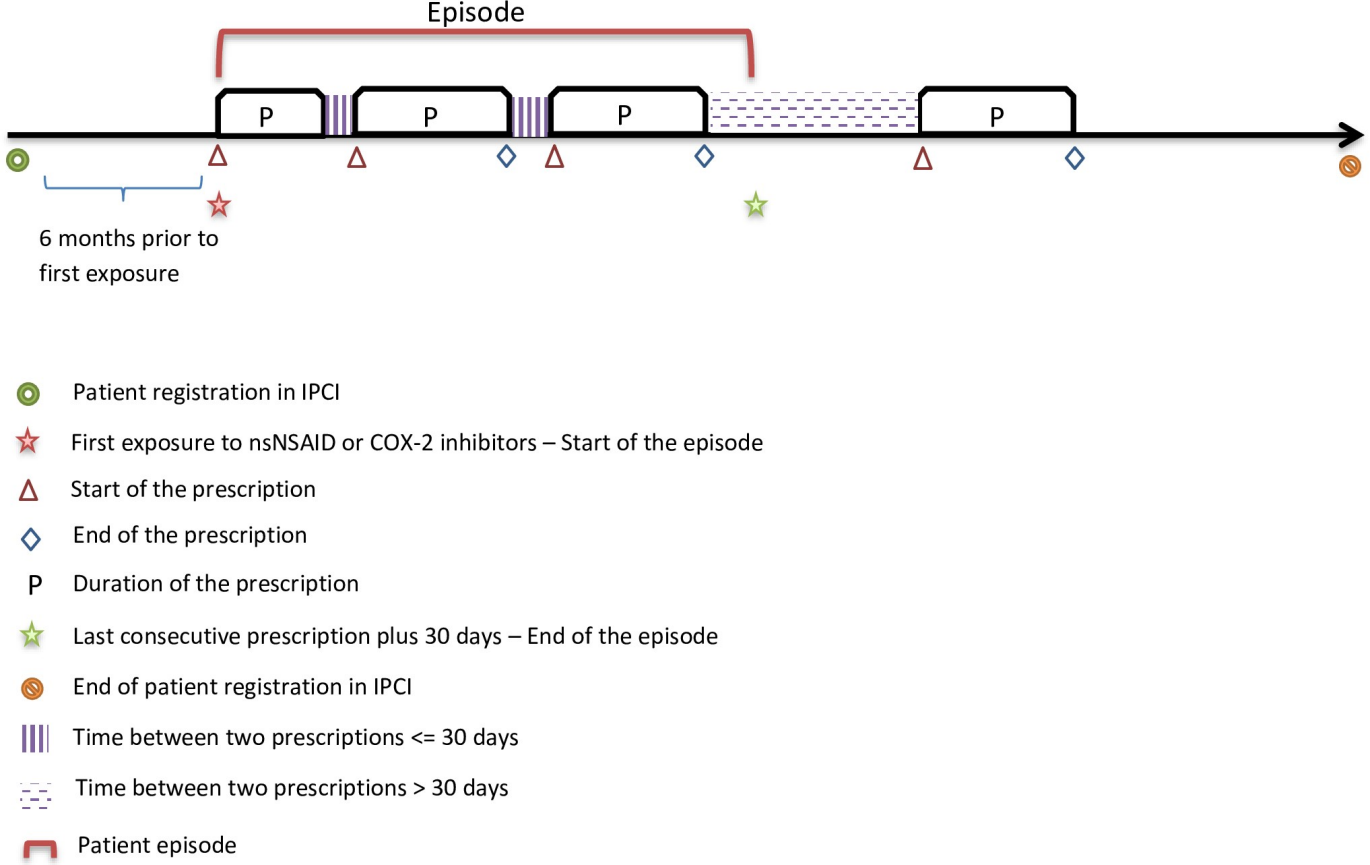
Episode selection.

We created a cohort of all new adult (≥18 years) users of NSAIDs between 1996 and 2013. Patients had to be enrolled for at least one year in the database in order to be eligible for cohort entry. ATC codes used for NSAID exposure are presented in S1 Table. Within the NSAID cohort we created episodes of ‘new’ NSAID use according to the following criteria: (a) at least six months of data available before NSAID exposure, (b) no prescription of any nonselective NSAID or selective COX-2 inhibitor in the previous six months (c) no mentioning of drug names, in the free-text, corresponding with NSAID-related ATC codes in the previous six months. The duration of a prescription was calculated by dividing the prescribed quantity by daily dose regimen. An NSAID episode continued when consecutive NSAID prescriptions started before or within 30 days of the end of the duration of the previous prescription. The end of the episode was defined as the end of the last NSAID prescription (see Fig 1). Episodes were classified as an nsNSAID or COX-2 inhibitor episode based on the first prescription in that episode being an nsNSAID or a COX-2 inhibitor, respectively. If a patient switched between exposure (from COX-2 inhibitor to nsNSAID or vice versa), the duration of the NSAID episode was ended at the switch of the exposure. A patient could have multiple NSAID episodes, but only if the above-mentioned criteria were met.

### Selection of upper gastrointestinal bleeding patients

Within the cohort of new NSAID users we identified all potential subjects who experienced an upper gastrointestinal bleeding (UGIB) via an automated search [26]. UGIB was defined as all forms of ulcer complications such as bleeding, perforation, or obstruction. The entire medical record of all potential UGIB patients was extensively reviewed to ensure the diagnosis and the date of onset. Any other cause of UGIB (such as variceal bleeding or Mallory Weiss bleeding) was excluded. The date of UGIB was determined as the date of first mentioning of symptoms leading to the UGIB diagnosis or if this date was unknown, the date of diagnosis.

### Propensity score model

A propensity model was fitted using all information (structured and unstructured) in the EHR. To reduce the number of potential variables we first converted all text to lowercase after which we removed special characters, words not starting with a letter or a digit, stop words (such as *de*, *het–*the article *the* in English), and punctuation. All unique words (also known as unigrams) in the 6 months prior to cohort entry were extracted and used as textual features (potential covariates). This approach is commonly known as bag-of-words (BoW) model. We tested two methods to limit the number of covariates that would be included in the regression. The first method generated models using covariates of which the frequency in the cohort was above a certain threshold, e.g., 1000 without any further selection. In the second method, we generated a model using covariates that were associated with the outcome. The chi-square test was used to select covariates that were statistically significantly associated with the outcome (p-value less than 0.05). Another propensity model (method 3) was added for comparison, where only the established confounders (i.e. age, sex, and the exposure to low-dose aspirin) were included in the propensity score model. We used patients’ prescription information to calculate exposure to low-dose aspirin.

**Fig 2:**
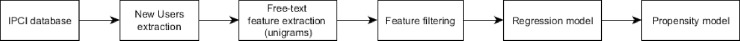
Flowchart showing the process of generating a propensity score model from unstructured free-text.

The selected features were subsequently used in a large-scale regularized regression using a LaPlace prior [27] with the hyper-parameter of 0.01 to construct a propensity model for each method. We experimented with several hyper-parameters and results of each are presented in the S4 Table. The advantage of using a regularized regression is that it can handle high-dimensional data. A flowchart depicting the process of propensity score model generation (for methods 1 and 2) is presented in Fig 2.

We used three-fold cross-validation [28] to evaluate the predictive accuracy of the models. The data set was randomly divided in three equally-sized subsets or folds. In three cross-validation runs, each time, the model was successively trained on two folds and tested on the third fold. For each cross-validation run, an area under the receiver operating characteristic curve (AUC) was calculated. The averaged AUC was used as the overall performance measure.

#### One-to-many propensity score matching

The propensity score that was generated in each of the two models was used to account for the preferential prescribing of COX-2 inhibitors to patients at high-risk of developing an UGIB [12]. In this study, we used the greedy one-to-many matching as described by Rassen et al. [29]:

1. For each COX-2 inhibitor cohort member the difference in PS with each nsNSAID users was computed
2. Starting with the lowest difference, each COX-2 inhibitor cohort member was matched with one nsNSAID cohort member. Once an nsNSAID user was matched, he or she was precluded from further matching. A caliper of 0.01 was used, meaning no matches were made if the difference in PS was greater than 0.01.
3. After all COX-2 inhibitor cohort members were matched with one nsNSAID cohort member, the process was repeated until all nsNSAID users were matched or there was no match possible.

The algorithm ensured that all COX-2 inhibitor cohort members were matched with at least one nsNSAID cohort member if such a match was available within the caliper.

### Statistical analysis

To estimate the risk of UGIB among COX-2 inhibitor users compared to nsNSAID users we calculated hazard ratios with their corresponding 95% confidence intervals (CIs) using Cox proportional hazard regression. We conducted the analysis for four datasets: 1) a crude comparison (unmatched, no propensity score); 2) matched on age (± 2 years) and adjusted for sex and exposure to low-dose aspirin, no propensity score; 3) matched on PS with covariate frequency above 1000 and then adjusted for age, sex, and exposure to low-dose aspirin; and 4) matched on PS with covariates having an association with the outcome and then adjustment for age, sex, and exposure to low-dose aspirin.

## Results

### NSAID cohort

From the source population of more than 1.8 million patients we identified 518,768 new users of NSAIDs based on ATC codes. We then processed the unstructured free-text in the entries of the new users to identify mentioning of drug names corresponding with NSAID-related ATC codes. In total, 36,188 new users were removed because either an nsNSAID or COX-2 inhibitor drug was mentioned in the free-text in the six months preceding first NSAID exposure. This resulted in 482,580 new NSAID users in the study cohort. Out of these, 459,701 (95%) were nsNSAID users and 22,879 (5%) were COX-2 inhibitor users.

Within the NSAID cohort we retrieved 11,994 potential UGIB patients. After reviewing the medical records we retained 1,048 UGIBs.

The average duration of episodes for initiators of COX-2 inhibitors was 94 days and 66 days for initiators of nsNSAIDs. Baseline characteristics of initiators of COX-2 inhibitors and nsNSAIDs are shown in Table 1. Most of the episodes of COX-2 inhibitors and nsNSAIDs were started after the year 2004.

**Table 1:** Baseline characteristics of initiators of selective COX-2 inhibitors or nsNSAIDs.

Characteristics	%	
	COX-2 initiators (n = 22,879)	nsNSAID initiators (n = 459,701)
Age (mean)	57.7	47.9
Male	36.5	43.2
Female	63.5	56.8
Exposure to low-dose aspirin	2.8	1.1
Age (years)		
< = 30	6.5	17.3
31–40	8.4	16.1
41–50	17.7	22.4
51–60	22.4	19.7
61–70	20.8	13.8
71–80	15.9	7.7
> 80	8.3	3.0
Calendar year of treatment initiation		
before 2003	0.1	10.8
2003	1.4	2.0
2004	3.1	1.9
2005	1.6	1.9
2006	1.5	1.3
2007	2.6	2.3
2008	7.3	6.7
2009	11.5	12.3
2010	15.6	16.4
2011	22.7	20.6
2012	30.7	22.7
2013	1.9	1.1
UGI risk factors		
Use of antiplatelets	6.3	3.2
Use of anticoagulants	3.2	1.3
Use of gastroprotective agents	23.4	11.8
Other comorbidities		
Dyspepsia	0.2	0.2
Smoking	0.5	0.5
Heart failure	0.4	0.2
Diabetes mellitus	0.5	0.3
Concomitant use of other medication		
SSRIs	4.4	3.3
Spironolactone	0.7	0.3
Calcium channel blockers	7.2	3.7

### Propensity model

In total, we extracted 2,762,326 covariates (i.e., unique words, out of almost 96 million words) from approximately 2.4 million entries in the 6 months prior to NSAID episodes from the medical records of 482,580 new NSAID users.

Table 2 shows the performance of the propensity models built using different covariates selection methods. The first model used all covariates with a frequency of 100 or more in the cohort, which resulted in 95,078 unique covariates entered into the model. Increasing the frequency to 1,000 resulted in a reduction of the number of covariates to 27,619. The number of covariates further reduced when frequency was increased to 5,000. The performance of the models in terms of their predictive accuracy was comparable. The predictive performance of the propensity model that was built using 3,650 covariates that had an association with the outcome according to the chi-square test. This resulted in an AUC of 70.59. The performance of the propensity model that included only the established confounders resulted in an AUC of 66.27. The number of covariates in the models however were only 111.

**Table 2:** Predictive performance of different propensity models.

**PS Model**		**Number of covariates**	AUC*
	Covariate filtered on frequency ≥ 100	95,078	72.27
Method 1	Covariate filtered on frequency ≥ 1,000	27,619	72.32
Method 2	Covariate filtered on frequency ≥ 5,000	11,699	72.17
	Covariates filtered using Chi-square test (independent of frequency)	3,650	70.59
Method 3	Only established confounders (age, sex, and exposure to low-dose aspirin)	111	66.27

* AUC, area under the receiver operating characteristic curve

### Risk of upper gastrointestinal bleeding

The crude hazard ratio of UGIB for COX-2 inhibitors compared to nsNSAIDs was 0.50 (95% 0.18–1.36) (Table 3). When matched on age, the hazard ratio of COX-2 inhibitor use compared to nsNSAID use was 0.36 (95% CI: 0.11–1.16). Further adjusting for sex and exposure to low-dose aspirin resulted in HR of 0.35 and 0.36 respectively. Matching on PS only, using one-to-many matching with a covariate frequency above 1,000, reduced the hazard ratio to 0.42 (95% CI: 0.13–1.38). Subsequent adjustment for age resulted in a hazard ratio of 0.36 (95% CI: 0.10–1.22). Matching on PS limiting to covariates that were associated to the outcome also provided a hazard ratio of 0.42 (95% CI: 0.13–1.39). Adjusting for age reduced the hazard ratio to 0.32 (95%: 0.09–1.09).

**Table 3:** Hazard ratios with 95% confidence intervals (CI) comparing COX-2 inhibitors with nsNSAIDs for different matching strategies and adjustments.

**Matching**	**Adjustment**	**Hazard ratio**	**95% CI**
**Unmatched**	None	0.50	0.18–1.36
**Age**	None	0.36	0.11–1.16
	Sex	0.35	0.11–1.18
	Sex, Aspirin	0.36	0.11–1.18
**Propensity Score (covariate frequencies > = 1000)**	None	0.42	0.13–1.38
	Age	0.36	0.10–1.22
	Sex	0.39	0.12–1.30
	Age, Sex	0.35	0.16–1.25
	Sex, Aspirin	0.39	0.12–1.32
**Propensity Score** **(covariates based on** **association test)**	None	0.42	0.13–1.39
	Age	0.32	0.09–1.09
	Sex	0.43	0.13–1.42
	Age, Sex	0.31	0.09–1.08
	Sex, Aspirin	0.43	0.13–1.42
	Age, Sex, Aspirin	0.31	0.09–1.10

The top-25 covariates, in terms of their weights (beta values), from both propensity score models are presented in the S2 and S3 Tables.

## Discussion

In this study, we generated a propensity model using unstructured information from EHRs. We tested different methods to construct this and demonstrated the feasibility to do so as well as its performance. Since electronic health records are now widely available for secondary use, we need to develop methods and test performance of these methods for use in epidemiological evaluations such as drug effects.

Our method to generate a propensity score model is substantially different from the high-dimensional propensity score (hd-PS) approach proposed by Schneeweiss et al [20]. The hd-PS algorithm that was developed for claims data uses structured information such as diagnostic codes, in-patient procedure codes, and drugs dispensed. In each identified data dimension, the highest ranked codes are selected to enter in the hd-PS model. The use of two-word free-text phrases in addition to the structured information has also been positively evaluated in the context of hd-PS models [22]. Our method is different since we used as the basis all unstructured text to generate propensity models, using a large-scale regularized regression, without pre-identified data dimensions. Several methods other than logistic regression such as data-adaptive and classification trees have been proposed for fitting a propensity model [30]. To reduce the number of ‘meaningless’ features, we needed various textual data cleaning steps. We subsequently extracted all unigrams from the cleaned free-text, which served as potential covariates. Here we applied different approaches, to look at the impact of our choices. In the first method, the most-frequent covariates in the cohort were selected to enter the propensity score model. Since the covariates were selected merely on the basis of their frequency in the cohort, this method is prone to include covariates that may actually be instrumental variables. Instrumental variables have an association with the exposure but not with the outcome except through their effect on exposure. If covariates are included that are not true confounders, the variance increases and sometimes a small amount of bias may be introduced [31–34]. In order to mitigate the potential to include covariates that are instrumental variables we included covariates with a significant association with the outcome to the propensity score model in the second method we applied [31].

We used three-fold cross-validation to evaluate the predictive performance of exposure to nsNSAID or Coxib for each generated PS model. In the first method where covariates were selected based on their frequency, increasing the frequency threshold for covariate selection reduced the number of covariates that entered into the propensity score model but the performance of the models was still comparable. This suggests that the performance of the models was mostly based on a few covariates with high occurrence in the text. Reducing the number of covariates reduced the computation time needed to fit the model. By selecting covariates with an association with the outcome, we significantly reduced the total number of covariates without greatly affecting the performance. The propensity models generated using covariates with only high frequency in the cohort performed better than the one where association with the outcome was verified. This may be due to the presence of some instrumental variables which can result in an increase in predictive performance [30]. We used another propensity model for the comparison purposes where only the established confounders age, sex, and exposure to low-dose aspirin were included. The predictive performance of this model was lower than the other two models which were generated from the free-text covariates. The second method, where covariate association with the outcome was verified, showed large decrease in the hazard ratios after further adjustments. Whereas previous studies have constructed the hd-PS with structured information, such as ICD and READ codes across different data dimensions in different sources [19–21,35], large proportions of information may be unstructured. We showed that this unstructured free-text can be used to construct propensity models. Initially, the new user cohort was created based on the prescription tables containing ATC codes. A high number of removals (7%) from the cohort based on the drug mentioned in the free-text indicates the importance of processing unstructured free-text instead of only relying on the structured information.

Our study also has several limitations. First, by including covariates based on their frequencies we might have selected covariates that are not necessarily related to the outcome or the exposure, which could introduce bias [18,36]. Second, since we only used unigrams, covariates like ‘*congestive heart failure’* cannot be recognized as such. Instead it will be recognized as individual words ‘*congestive’*, *‘heart’*, and ‘*failure’*, which might lead to over- and underestimation of some covariates. Like previous studies using hd-PS methods, we also used the known association between NSAIDs and UGIB as an example. It is unclear whether our findings regarding the PS generated from unstructured free-text apply to other treatment-outcome pairs. Since the PS algorithm in general relies on the information present in the cohort, a similar approach using a different data set might have different results even when using known example of NSAID-UGIB.

The majority of COX-2 inhibitor episodes started after the year 2004, the period after the withdrawal of rofecoxib from the market because of cardiovascular risks [37]. This may explain the strong protective effect of COX-2 inhibitors in the crude analysis which we would expect, but is different from previous observational studies that were done more closely to the introduction of coxibs [19–21,35]. Since most of our patients started after the contra-indications were introduced, channeling towards high risk patients was less of an issue [38].

In conclusion, our study showed that PS models can be created using unstructured information in electronic healthcare records. We also showed that the PS model where covariates were filtered on their association with the outcome provide an improvement in adjustment for confounding. This is useful for database studies using a large amount of unstructured free-text as in EHRs. Better methods for extracting meaningful covariates from the free-text may be required for effective proxy adjustment via propensity scores.

## Supporting information

S1 Table List of ATC codes used for NSAID exposure assessment. (DOCX)

S2 Table Top 25 covariates by their weights selected by the regression model (covariates frequency > 1000). (DOCX)

S3 Table Top 25 covariates by their weights selected by the regression model (chi-square test). (DOCX)

S4 Table All tested hyper-parameters with AUCs for training and test sets. (DOCX)
