# Supplementary material for: Generating and evaluating a propensity model using textual features from electronic medical records
Source: PLoS One. 2019 Mar 4;14(3):e0212999. doi: 10.1371/journal.pone.0212999 (PMC6398864; doi:10.1371/journal.pone.0212999)
Supplement: S1 Table — (DOCX) [file pone.0212999.s001.docx]

S1 Table: List of ATC codes used for NSAID exposure assessment.

| **ATC code** | **Name** | **Type of NSAID** |
| --- | --- | --- |
| M01AA* | Butylpyrazolidines | non-selective NSAID |
| M01AB* | Acetic acid derivatives | non-selective NSAID |
| M01AC* | Oxicams | non-selective NSAID |
| M01AE* | Propionic acid derivatives | non-selective NSAID |
| M01AG* | Fenamates | non-selective NSAID |
| M01AH01 | Celecoxib | COX-2 inhibitor |
| M01AH03 | Valdecoxib | COX-2 inhibitor |
| M01AH04 | Parecoxib | COX-2 inhibitor |
| M01AH05 | Etoricoxib | COX-2 inhibitor |
| M01AH06 | Lumiracoxib | COX-2 inhibitor |

*all drugs from this group are included
