# Supplementary material for: Generating and evaluating a propensity model using textual features from electronic medical records
Source: PLoS One. 2019 Mar 4;14(3):e0212999. doi: 10.1371/journal.pone.0212999 (PMC6398864; doi:10.1371/journal.pone.0212999)
Supplement: S2 Table — (DOCX) [file pone.0212999.s002.docx]

S2 Table: Top 25 covariates by their weights selected by the regression model (covariates frequency > 1000)

| Rank | Unigram | Translation | Beta value |
| --- | --- | --- | --- |
| 1 | dh* | diakonesse huis / hospital | 0.565 |
| 2 | rrzit | blood pressure measurement in sitting position | 0.548 |
| 3 | nabehandeling | Follow up treatment | 0.532 |
| 4 | school | School | 0.430 |
| 5 | orthopaedisch | Orthopedic | 0.376 |
| 6 | specialistische | Specialistic | 0.360 |
| 7 | bacteri | Bacteria | 0.334 |
| 8 | tonsillen | Tonsils | 0.326 |
| 9 | acne | Acne | 0.319 |
| 10 | rfe* | reason for encounter | 0.312 |
| 11 | tonsillitis | Tonsillitis | 0.303 |
| 12 | bloedafname | Blood sampling | 0.287 |
| 13 | nvgb* | niet verschenen geen bericht / patient did not show up at consult/appointment and left no message | 0.285 |
| 14 | origineel | Original | 0.282 |
| 15 | menstruatie | Menstruation | 0.279 |
| 16 | arthroscopie | Arthroscopy | 0.241 |
| 17 | housenumber | house number | 0.238 |
| 18 | bloedbeeld | Complete blood test | 0.236 |
| 19 | waarneming | Observation | 0.225 |
| 20 | ref* | Reference / referral | 0.225 |
| 21 | abnormaal | Abnormal | 0.223 |
| 22 | n89 | ICPC Code N89 (Migraine) | 0.217 |
| 23 | glu* | glucose | 0.203 |
| 24 | assistent | Assistant | 0.202 |
| 25 | spastische | Spastic | 0.201 |

* Abbreviations, might have other meanings as well depending on the context.
