# Supplementary material for: Generating and evaluating a propensity model using textual features from electronic medical records
Source: PLoS One. 2019 Mar 4;14(3):e0212999. doi: 10.1371/journal.pone.0212999 (PMC6398864; doi:10.1371/journal.pone.0212999)
Supplement: S3 Table — (DOCX) [file pone.0212999.s003.docx]

S3 Table: Top 25 covariates by their weights selected by the regression model (chi-square test)

| Rank | Unigram | Translation | Beta value |
| --- | --- | --- | --- |
| 1 | rrzit | Blood pressure measurement in sitting position | 0.675 |
| 2 | dh* | diakonesse huis / hospital | 0.656 |
| 3 | school | School | 0.545 |
| 4 | izh* | ijselland ziekenhuis / hospital | 0.413 |
| 5 | rfe* | reason for encounter | 0.376 |
| 6 | tonsillen | Tonsils | 0.369 |
| 7 | acne | Acne | 0.357 |
| 8 | cvx* | cervix | 0.347 |
| 9 | declareren | Declare | 0.345 |
| 10 | menstruatie | Menstruation | 0.343 |
| 11 | tonsillitis | tonsillitis | 0.309 |
| 12 | ref* | Reference / referral | 0.306 |
| 13 | diak* | diakonesse huis / hospital | 0.295 |
| 14 | erythro | Erythrocyte | 0.264 |
| 15 | kindergeneeskunde | Pediatrics | 0.263 |
| 16 | bultje | Bump | 0.254 |
| 17 | exfoliatieve | exfoliation | 0.248 |
| 18 | intensieve | Intensive | 0.247 |
| 19 | arthroscopie | Arthroscopy | 0.240 |
| 20 | fp |  | 0.236 |
| 21 | asdrukpijn | Axial pressure pain | 0.234 |
| 22 | bevalling | Delivery | 0.234 |
| 23 | zwanger | Pregnant | 0.230 |
| 24 | regulair | Regular | 0.224 |
| 25 | zelfcontrole | Self-control | 0.214 |

* Abbreviations, might have other meanings as well depending on the context.
