# Supplementary material for: Generating and evaluating a propensity model using textual features from electronic medical records
Source: PLoS One. 2019 Mar 4;14(3):e0212999. doi: 10.1371/journal.pone.0212999 (PMC6398864; doi:10.1371/journal.pone.0212999)
Supplement: S4 Table — (DOCX) [file pone.0212999.s004.docx]

S4 Table: All tested hyper-parameters with AUCs for training and test sets

| Parameters | | Training | Testing |
| --- | --- | --- | --- |
| Variance (-V) | Convergence threshold (-e) | AUC | AUC |
| default | 0.0005 (default) | 98 | 57 |
| 1 | 0.000001 | 88.3 | 68 |
| 0.2 | 0.000001 | 83.1 | 71 |
| 0.1 | 0.000001 | 81 | 71.6 |
| 0.01 | 0.000001 | 76.1 | 72.3 |
| 0.001 | 0.000001 | 73.2 | 71.3 |
| 0.0001 | 0.000001 | 70.5 | 69.2 |
